# *Clostridium difficile* Infection, Colorado and the Northwestern United States, 2007^1^

**DOI:** 10.3201/eid1806.111528

**Published:** 2012-06

**Authors:** Jennifer L. Kuntz, Eric S. Johnson, Marsha A. Raebel, Amanda F. Petrik, Xiuhai Yang, Micah L. Thorp, Steven J. Spindel, Nancy Neil, David H. Smith

**Affiliations:** Kaiser Permanente Northwest, Portland, Oregon, USA (J.L. Kuntz, E.S. Johnson, A.F. Petrik, X. Yang, M.L. Thorp, S.J. Spindel, D.H. Smith);; Kaiser Permanente Colorado Institute for Health Research, Denver, Colorado, USA (M.A. Raebel);; University of Colorado School of Pharmacy, Denver (M.A. Raebel);; Decision Research, Eugene, Oregon, USA (N. Neil)

**Keywords:** Clostridium difficile, infection, incidence, bacteria, Colorado, United States

## Abstract

To determine the incidence of *Clostridium difficile* infection during 2007, we examined infection in adult inpatient and outpatient members of a managed-care organization. Incidence was 14.9 *C. difficile* infections per 10,000 patient-years. Extrapolating this rate to US adults, we estimate that 284,875 *C. difficile* infections occurred during 2007.

*Clostridium difficile* infection is a major source of illness in the United States (*1*). Population-based estimates of its incidence tend to include only subsets of infections defined by the setting of *C. difficile* acquisition (*2**–**4*) or are from patient populations outside the United States (*5**–**9*). These studies (*2**–**9*) offer useful data for control measures but do not help clinicians and policy makers understand the population-based incidence of *C. difficile* infection. To determine the incidence of *C. difficile* infection during 2007, we estimated the incidence of *C. difficile* infection among members of 2 Kaiser Permanente health plans and extrapolated our incidence estimate to the US adult population.

## The Study

We identified *C. difficile* infections during January 1–December 31, 2007, among Kaiser Permanente Colorado and Kaiser Permanente Northwest members >20 years of age. The health plans had a combined membership of ≈900,000 on any given day during 2007. We collected patient membership, demographic, and clinical data using electronic databases. *C. difficile* infections were identified through International Classification of Diseases, 9th Revision (ICD-9), code 008.45 (“Intestinal infection due to *C. difficile*”) recorded during an inpatient or outpatient health care visit or a positive *C. difficile* toxin test result. To increase the likelihood that cases were symptomatic, we further required that positive toxin test results be associated with dispensation of metronidazole or vancomycin in the outpatient pharmacy in the 7 days before or after a positive test result. Specimens were reported as negative or positive on the basis of results from a Meridian Premier Toxin A/B enzyme immunoassay (Meridian Bioscience, Cincinnati, OH, USA).

A *C. difficile* infection was considered incident if the patient did not have a history of a *C. difficile* diagnosis, a positive toxin test result, or an outpatient prescription for vancomycin or metronidazole in the previous 180 days. To ensure cases were incident and to collect baseline characteristics, patients with *C. difficile* infections were required to have continuous membership and prescription drug coverage for 1 year before the date of *C. difficile* infection.

We calculated the total incidence of *C. difficile* infection as the number of incident cases among persons >20 years of age per 10,000 person-years of observation. Age- and sex-specific incidence rates were also calculated. A patient could have had >1 incident *C. difficile* infections if they occurred >180 days apart. Denominator data were based on duration of membership for persons >20 years of age with continuous membership and prescription drug coverage for 1 year before July 31, 2007. To project the national incidence of *C. difficile* infection, we applied pooled, age-specific incidence, and sex-specific incidence estimates to the 2007 US population. As a sensitivity analysis, we also provided the incidence projection for US whites because earlier surveys of members showed a predominantly (90%) white membership, and race data were unavailable for a substantial proportion of members.

We identified 870 incident *C. difficile* infections among members >20 years of age in 2007; a total of 473 (54%) of 870 *C. difficile* infections were identified among outpatients. Overall incidence was 14.9 *C. difficile* infections per 10,000 patient-years; age-specific incidence rates ranged from 2.4 infections per 10,000 patient-years for men 20–29 years of age to 87.1 infections per 10,000 patient-years for men >80 years of age (Table). On the basis of these age- and sex-specific rates, we estimated that 284,875 *C. difficile* infections occurred among the overall US population >20 years of age. We also estimated that 241,815 infections occurred among US whites (Table).

**Table:** Incidence of *Clostridium difficile* infection among members >20 years of age of Kaiser Permanente Colorado and Northwest, USA, with projections to US white and US total populations, 2007*

Sex/age, y	Kaiser Permanente populations			Projected no. US infections	
	Incident infections, no.	Incidence rate* (SE)		Whites	Total
Female					
20–29	12	3.1 (0.91)		4,920	6,383
30–39	31	6.1 (1.09)		9,399	12,186
40–49	45	7.2 (1.1)		13,079	16,386
50–59	80	11.5 (1.3)		18,892	23,046
60–69	96	21.3 (2.2)		23,340	27,681
70–79	119	42.0 (3.8)		32,141	37,503
>80	140	79.5 (6.7)		42,559	48,015
Male					
20–29	8	2.4 (0.85)		3,969	5,009
30–39	18	3.9 (0.93)		6,318	7,871
40–49	29	5.2 (0.96)		9,274	11,321
50–59	62	10.1 (1.3)		16,242	19,359
60–69	67	16.7 (2.0)		16,747	19,355
70–79	73	31.1 (3.6)		18,994	21,780
>80	90	87.1 (9.2)		25,941	28,980
All	870	14.9 (0.5)		241,815	284,875

*Per 10,000 patient-years.

## Conclusions

Our study identified new infections among this managed care population in 2007 and estimated the occurrence of new infections within the US population. Our approach differed from those of previous reports that were based on individual medical institutions, hospital discharge databases, or voluntary surveillance in targeted populations and geographic areas (*10**–**13*). We differentiated between incident and prevalent infections. Identification of incident infections is needed to understand the causes and predictors of infection and to develop clinical interventions to prevent infection. Because we also identified *C. difficile* infection among inpatients and outpatients, our estimates more fully account for total *C. difficile* infection and provide a foundation for studying the potential spread of *C. difficile* between ambulatory and hospitalized persons.

Although different objectives and methods make direct comparisons difficult, we found that the incidence of *C. difficile* infection among the Kaiser Permanente population studied (14.9 infections/10,000 patient-years) did not differ substantially from the 11.2 *C. difficile*–associated hospitalizations among adults per 10,000 population identified in the 2005 Nationwide Inpatient Sample (*14*). Our estimate of 240,000–285,000 incident *C. difficile* infections in the US adult population in 2007 is comparable with the results of an extrapolation by Campbell et al., which found that on the basis of health care–facility surveillance data, 333,000 initial and 145,000 recurrent *C. difficile* infections might have occurred nationwide in 2006 (*15*). Although previous reports of inpatient encounters resulting from *C. difficile* infection or total *C. difficile* infections are needed to describe the impact of *C. difficile* infection on the health care system, our identification of infections in inpatient and outpatient health care settings may provide a more accurate estimate of *C. difficile* infection incidence.

Many discharge databases do not include longitudinal patient identifiers; even though these databases can identify diagnoses during hospitalizations, they cannot link hospitalizations to specific patients. Thus, multiple hospitalizations for recurrent or refractory *C. difficile* infection would count as multiple infections, whereas we followed-up patients over time to ensure that only incident infections were counted. Furthermore, although we relied on ICD-9 codes to identify cases among inpatients, we also used *C. difficile* toxin tests, treatment for *C. difficile* infection, and *C. difficile*–related health care encounters to identify infections in outpatients. We could have missed infections in inpatients if the ICD-9 code for *C. difficile* was absent; however, our use of toxin test results and pharmacy dispensing records likely resulted in more accurate and complete identification of *C. difficile* infection among outpatients. In fact, we found that >95% of patients with positive toxin test result had a treatment-dispensing or ICD-9 code for *C. difficile* infection.

Our data might overestimate or underestimate the incidence of *C. difficile* infection or affect the interpretation of our results in 4 ways. First, our population was predominantly white. Although we are unaware of any evidence that *C. difficile* infections occur disproportionately by race or ethnicity, we projected our incidence rate to the entire and white-only US populations to acknowledge the distribution of race in the study population. Second, our insured population could be healthier than the US population. Third, Kaiser Permanante has policies and procedures that promote judicious prescription of antimicrobial drugs and effective infection control and prevention. Collectively, a healthy population, health plan policies, and clinician awareness might result in fewer *C. difficile* infections among this population than is observed in other health care settings. Fourth, toxin tests are imperfect, potentially leading to overestimation of incidence.

Our study provides population-level estimates of *C. difficile* infection in inpatients and outpatients. However, more efficient and timely methods for identifying and reporting *C. difficile* infection are needed to further improve understanding of the epidemiology of *C. difficile* infection and the interventions necessary to prevent them.
